# Improving the colorectal cancer care pathway via e-health: a qualitative study among Dutch healthcare providers and managers

**DOI:** 10.1007/s00520-023-07653-2

**Published:** 2023-03-06

**Authors:** Liza van Deursen, Rosalie van der Vaart, Eva E. Alblas, Jeroen N. Struijs, Niels H. Chavannes, Jiska J. Aardoom

**Affiliations:** 1 Department of Quality of Care and Health Economics, Center for Nutrition, Prevention, and Health Services, National Institute for Public Health and the Environment, Bilthoven, The Netherlands; 2 National eHealth Living Lab, Leiden, The Netherlands; 3 Department of Public Health and Primary Care, Health Campus The Hague, Leiden University Medical Center, The Hague, The Netherlands; 4 Department of Public Health and Primary Care, Leiden University Medical Center, Leiden, The Netherlands

**Keywords:** E-health, Digital, Colorectal, Cancer, Quadruple Aim, Quality of care

## Abstract

**Purpose:**

This study aims to identify improvement opportunities within the colorectal cancer (CRC) care pathway using e-health and to examine how these opportunities would contribute to the Quadruple Aim.

**Methods:**

In total, 17 semi-structured interviews were held (i.e., nine healthcare providers and eight managers involved in Dutch CRC care). The Quadruple Aim was used as a conceptual framework to gather and systematically structure the data. A directed content analysis approach was employed to code and analyze the data.

**Results:**

Interviewees believe the available e-health technology could be better exploited in CRC care. Twelve different improvement opportunities were identified to enhance the CRC care pathway. Some opportunities could be applied in one specific phase of the pathway (e.g., digital applications to support patients in the prehabilitation program to enhance the program’s effects). Others could be deployed in multiple phases or extended outside the hospital care setting (e.g., digital consultation hours to increase care accessibility). Some opportunities could be easily implemented (e.g., digital communication to facilitate treatment preparation), whereas others require structural, systemic changes (e.g., increasing efficiency in patient data exchanges among healthcare professionals).

**Conclusion:**

This study provides insights into how e-health could add value to CRC care and contribute to the Quadruple Aim. It shows that e-health has the potential to contribute to the challenges in cancer care. To take the next step forward, the perspectives of other stakeholders must be examined, the identified opportunities should be prioritized, and the requirements for successful implementation should be mapped out.

**Supplementary Information:**

The online version contains supplementary material available at 10.1007/s00520-023-07653-2.

## Introduction

Colorectal cancer (CRC) is the second most common cancer in Europe [1, 2] and the second deadliest cancer worldwide, with more than 900,000 reported deaths in 2020 due to CRC [2]. However, the survival rate is increasing due to earlier detection as a result of better screening and more extensive treatment options [3, 4]. Similar to most other healthcare areas, CRC care faces significant challenges. First, cancer incidence rates are expected to double worldwide in the coming decades due to substantial demographic changes, among which a growing and aging population [5, 6]. Second, healthcare needs, expenditures, and personnel shortages will increase [7]. Global nursing shortages, for example, are expected to rise to 12.9 million in 2035 [8]. Third, as treatment options expand, CRC care can become increasingly complex [9].

In the context of these challenges, e-health, defined as “the use of information and communications technologies (ICT) in support of health and health-related fields” [10], is considered a possible way to optimize health service delivery (Cowie et al., 2013; Kimble, 2015; Moerenhout et al., 2018). E-health can increase healthcare delivery efficiency, decrease healthcare expenditures, and enhance care quality [11], for example, through improved patient-provider communication, better symptom management, and increased patient engagement [12]. Potentially, e-health can change CRC care delivery in different ways. Telemonitoring, for example, can facilitate tracking a cancer patient’s health status for early relapse detection, thereby preventing complications and reducing healthcare costs [13–15].

Although many e-health applications are already available and used experimentally, the structural integration of e-health into regular healthcare is challenging [16, 17]. To facilitate the integration of e-health in daily practice, the perspective of healthcare managers and healthcare providers on how e-health can add value to daily practice should be considered, as well as how this contributes to improving care quality, population health, and staff experience and decreasing per capita costs often referred to as the Quadruple Aim [17, 18].

Therefore, this study aimed to explore healthcare providers’ and managers’ views on improvement opportunities within the CRC care pathway using e-health and to examine how this would contribute to the Quadruple Aim.

## Materials and methods

### Design, sampling, and recruitment

Semi-structured online interviews were held with Dutch healthcare providers (i.e., nurses and colorectal surgeons) and managers (i.e., care directors, policy officers, or managers) working in a hospital (i.e., the medical specialist care setting) and being involved in the care of CRC patients.

A purposive sampling method [19] was used, ensuring heterogeneity concerning (1) healthcare providers versus healthcare managers, thereby including perspectives of both care executors and managers; (2) type of healthcare provider profession, as different professions are involved in different parts of the care pathway; and (3) academic versus non-academic hospitals, as they tend to vary in the care they deliver for CRC patients and how they use e-health. The sample size was not predetermined: interviews with healthcare providers and managers were held until data saturation was reached (i.e., when new incoming data produced little or no further information to address the research question). Interviewees were recruited through network organizations and social media (i.e., LinkedIn).

The study was declared to not fall within the scope of the Dutch Medical Research Involving Human Subjects Act by the clinical expertise center of the Dutch National Institute for Public Health and the Environment (VPZ-552). The research protocol was reviewed by a scientific advisory committee consisting of representatives of the integrated cancer center in the Netherlands, the Dutch Association of Hospitals, the Federation of Medical Specialists, and the Dutch Open University, as well as by two nurses involved in the CRC care. The interview protocol was pilot tested with another CRC care nurse. All interviewees provided written informed consent and were fully informed of the study’s objectives and characteristics.

### Data collection

Three researchers performed individual online interviews (L. v. D., E. A., and J. A.). The protocol followed a semi-structured format, allowing the moderator to change the order of the questions or clarify questions if needed [20]. The main interview topic was the potential and opportunities of e-health in improving the CRC care pathway. In addition, questions were asked about the current use of, and experiences with, e-health for CRC patients at the start of the interview to get a general idea of the extent to which healthcare professionals and managers already use e-health and what their general attitude is towards the use of e-health for CRC care. Examples of probing questions were (1) To what extent do you use e-health in your daily work? and (2) Where do you see opportunities to better organize care within the care pathway, and how can e-health contribute to this?

The Dutch medical specialist care for CRC patients is organized into care pathways [21], usually including the following phases: referral, diagnosis, treatment, aftercare, and (in some cases) palliative care [22]. The care pathway was presented during the interviews. More details are presented in Table 1 [23, 24].

**Table 1:** Overview of the different phases of the CRC care pathway

Phase	Description
Referral	Patients are referred to a hospital-based on national population screening test results or if a general practitioner suspects CRC
Diagnosis	The patient undergoes one or more tests to locate the tumor, determine the type and growth rate, and determine whether there are metastases. An intake interview and an endoscopy or an examination of the large intestine using an endoscope are usually conducted
Treatment	Based on the diagnosis, treatment options are discussed with the patient. Examples of treatment options are surgery or chemotherapy. Patients often follow a pre-habilitation program to prepare for surgery (e.g., physical training, advice on nutrition, and mental support)
Aftercare	When (part of) the treatment is completed, patients are supported in their recovery, and any complications are monitored. Referral (i.e., to social workers or physiotherapists) can occur based on physical and psychological health monitoring
Palliative care	Patients who cannot recover from CRC receive care to optimize quality of life. The care consists of, among other things, pain relief and (psycho) social support. Advance Care Planning (ACP) is used to discuss the wishes and needs of patients with a healthcare provider

Before the interview, each interviewee received an information sheet with the World Health Organization’s definition of e-health (i.e., “the use of information and communication technology in support of health and health-related fields”) [25] and some examples of e-health categories. We developed the categories based on a framework of Nictiz [26], which classifies e-health according to the dominant technology used. We adapted these to relevant categories of technology and digital health services for CRC care. We used the following categories: digital communication, electronic data exchange, telemonitoring, self-monitoring, online information services, personal health environment, decision support software, and patient portals. The information sheet was also shown during the interview. The conceptual framework (see below) and the care pathway phases were also shown. The interviews had a duration of approximately 60 min. All interviews were video-recorded and transcribed verbatim using a transcription service.

### Conceptual framework

The Quadruple Aim framework was used as a conceptual framework in this study. The Quadruple Aim framework describes an approach to improve health system performance by focusing on four pillars: (1) increasing the quality of care, (2) increasing population health, (3) improving staff experience, and (4) reducing per capita costs [18]. We presented the four aims to the interviewees as a starting point for them to consider improvement opportunities and to systematically analyze and summarize the results.

### Data analysis

A directed content analysis approach was employed using both a priori codes based on topics from the interview guide and codes emerging during analysis [27]. After the first two interviews were coded, three researchers (L. v. D., E. A., and R. v. d. V.) reviewed, refined, and consolidated a preliminary coding book, which was shared and discussed with the entire research team. Two researchers (L. v. D. and E. A.) independently coded all interviews. Differences were discussed until a consensus was reached. Data saturation was reached concerning the identified themes. Data analyses were conducted in MAXQDA 2022 software [28].

## Results

### Interviewees’ demographics and earlier use of e-health

An overview of the demographic information of the interviewees (*N* = 17) can be found in Table 2. The mean age of the interviewees was 45 ± 8 years (range 32 to 62 years). We interviewed nine healthcare providers (nurses, nurse specialists, gastroenterologists, and colorectal surgeons) and eight managers (care directors, managers, or policy officers).

**Table 2:** Characteristics of interviewees (*N* = 17)

Characteristic	*n*	(%)
Sex		
Male	3	(18)
Female	14	(82)
Profession		
Nurse	3	(18)
Nurse specialist	3	(18)
Gastroenterologist or colorectal surgeon	3	(18)
Care director, manager, or policy officer	8	(47)
Type of hospital		
Academic	7	(41)
Non-academic	10	(59)

Since the start of the COVID-19 pandemic, the use of e-health technology has increased. Interviewees reported that various types of e-health are already being used but that current use often can be considered suboptimal. See Supplementary file 1 for an overview of interviewees’ usage of and experiences with different e-health categories.

Generally, the interviewees see the potential of e-health to make healthcare more patient-oriented, efficient, and flexible (i.e., independent of place and time). At the same time, interviewees mentioned that current societal and demographic changes (e.g., aging society, personnel shortages) create urgency and pressure to change healthcare delivery. If not, the healthcare system in its current form is no longer sustainable. Some interviewees are, however, concerned about some patient groups (i.e., elderly patients) being unable to use e-health. Finally, it is noted that healthcare professionals are not always motivated to use e-health (e.g., because they do not find it suitable for their patients).

### Improvement opportunities in the CRC care pathway using e-health

The most significant improvement opportunities in the CRC care pathway with the support of e-health mentioned by the interviewees are described below. We explain per opportunity what it would look like according to the interviewees. The opportunities are presented for each phase of the CRC care pathway. Some improvement opportunities are not specific to one of the care pathway phases: they apply to multiple phases of the care pathway and/or are not limited to the hospital care setting. Table 3 explains how each opportunity contributes to the Quadruple Aim according to the interviewees, and a quotation is provided for illustration.

**Table 3:** Description of how each improvement opportunity contributes to the Quadruple Aim according to the interviewees

Phase	Improvement opportunity	QA^1^	Quotation
Referral and diagnosis	**1. A digital intake for an endoscopy could contribute to better-prepared patients.** Patients are more likely to remember the information as they can re-read or re-check it, which could contribute to better preparation for the endoscopy. It would also save healthcare providers time and reduce costs	Q, S, C	“I think we actually explain that less well than when you watch a video. And, of course, people can also watch the video again. We now explain that in an intake consultation […]. It has become quite a chore for us. I think that much information is lost with patients and that you can do that better digitally.” [Interview 12, healthcare provider]
Treatment	**2. Online information services could better inform patients about treatment options.** Patients tend not to remember all the information provided during a consultation and have a need to re-read and reconsider information. The services could help patients to make a better-substantiated treatment choice, increasing patients’ control and facilitating joint decision-making. The treatment choice and related care would better fit patients’ needs and wishes	Q	“You know, a lot is often discussed in a consultation, and a decision must be made. So, I think we can inform patients much better by getting them to think about this [treatment options] in advance. And you can fill in a decision aid: how am I in life? What is my quality of life now? And those are the things that are not offered in a regular consult.” [Interview 12, healthcare provider]
	**3. Digital communication and online information services could facilitate treatment preparation**. Digital consultations could increase flexibility, and relatives could join easier. Online information and a chat service could give patients faster and easier access to information and allow them to ask questions whenever they need clarification. This could improve patients’ treatment preparation, and lead to caregivers spending less time answering questions once patients are in the hospital	Q, S, C	“[Digital videos contribute to] more control for the patient and a well-informed patient. And a better-supported patient because the conversations we have allow us to zoom in much more on what it means for you [as a patient] and what support you need. Ultimately, I also think the experience of the caregivers improves. Because now we have a lot to say and a lot to do. Ultimately, we feel we are running out of time. I can pay attention again to the important things and that must be done in person.” [Interview 5, manager]
	**4. A digital application could support patients in the prehabilitation process.** Patients’ feeling of being in control could increase. It could also enable caregivers to signal more quickly when the prehabilitation is not going well, enabling them to intervene earlier, possibly leading to better program effects. This could enhance patients’ recovery, reduce the risk of complications, and lower costs	P, Q, C	“And with that pre-habilitation, you have to do something with it digitally. Because that is so complex, there are so many care providers involved. It is almost impossible for a case manager to keep track of that per patient. So then you want to put more control in the hands of that patient and that he knows well, okay, where am I? What else do I have to do? There must be a digital environment for that.” [Interview 3, manager]
Aftercare	**5. Remote wound and stoma care could reduce hospital visits and consultations**. The possibility of uploading images to the patient portal and getting responses from care providers could enable patients to be discharged early, even when home care has not been arranged. Care providers could provide home care with timely and personal assistance through video calling, and intervene more quickly in the event of complications, reducing costs	Q, C	“We are often called by home care, for example, because there are problems with the stoma (…). We have stoma nurses in the hospital for that. They also often work with images forwarded from the home care organization. However, it would also be nice if, for example, they can give straightforward advice using video calling.” [Interview 9, healthcare provider]
	**6. Patient telemonitoring in the post-operative phase could increase the chance of early complication recognition.** This could prevent further patient deterioration. Sometimes, it could mean patients can be discharged earlier, saving costs. Daily monitoring of patient’s health values from within the hospital could relieve staff, improving their experience	P, Q, S, C	“I think we can improve the quality of care by monitoring patients at home, especially during the operative phase (…). So that we identify the risk much faster than we do now if we only see the patient in the outpatient clinic after ten days.” [Interview 15, manager]
	**7. Telemonitoring for prolonged patient monitoring after treatment. **This could lead to faster identification of complications, cost savings, and better quality of care. Digital follow-up consultations could decrease hospital visits	P, Q, C	“Why does a patient have to come to the hospital and tell how he is doing? That’s very much before Covid, shall we say.” [Interview 7, manager]
Palliative care	**8. Digital information about physical and psychological complaints could support patients.** The information could enhance patients’ knowledge about whether common complaints are expected or deviate from what is expected in this phase, which could prevent further complications and hospital visits. Also, patients would not come to the hospital unnecessarily because they are better informed about what is abnormal and what is not, increasing the quality of care	P, Q, C	“And then someone comes in with fluid in his stomach, those are always those crazy things that you think; well, I’m doing it all so well, and you know our palliative consultant, and you still have low-threshold contact with your case manager and your head practitioner. And yet you eventually report to the emergency room because you can’t anymore, and you are in such pain and have many liters of fluid in your abdomen.” [Interview 12, manager]
	**9. A digital Advance Care Planning questionnaire could support joint decision-making.** The digital questionnaire could also result in more effective consultations and care better suited to patients’ palliative care needs	Q, S	“Joint decision-making simply means that you have a conversation with the patient. And I think that palliation should also be discussed at an early stage. When you ask a patient; what are the most important treatment goals for you? And if you structurally offer questionnaires to patients from the very first moment, you can also see where changes take place. And then you also have an opening to talk to the patient.” [Interview 1, manager]
Care pathway phase transcending	**10. Digital consultation hours or a chat could unburden staff.** Healthcare providers would receive fewer patient calls during or in-between care activities, improving staff experience. Care could be better accessible for patients with (sensitive) questions, and unnecessary hospital visits could be avoided, reducing costs	Q, S, C	“We are now called on the ward by patients who have been discharged for several days. That, of course, goes through our daily tasks, so if that can be done in a more targeted way, for example, by allowing a patient to ask a question or make a report in the patient portal, then a response will be generated faster. And there is immediately someone who can do something with it.” [Interview 9, healthcare provider]
	**11. Digital questionnaires in preparation for a consultation could facilitate patients to report complaints or ask questions.** Healthcare providers could ask more specific questions during the consultation. If the questionnaire shows everything is going well, some consultations could become unnecessary. The gathered data could also be used for research	Q, C	“Who do you live with and what kind of house do you live in and have you already had contact with them and… Those are things that we very often ask twice […]. But many things, in my opinion, could simply be left to the patient.” [Interview 10, healthcare provider]
Hospital care transcending	**12. More efficient digital data exchange improves the availability of patient data for healthcare providers and patients.** This could lead to cost savings as fewer additional consultations are required to obtain patient information, reducing healthcare providers’ workload. It could contribute to a better transfer of patient data between care providers and more care quality transparency at a national level	Q, S, C	“How annoying is it that you have a sick patient in bed but do not have the correct data available because images still have to be scanned? Or the medication list has not come across. And that’s frustrating, and it all takes time, and it’s at the expense of the patient’s health. And, yes, this also increases the healthcare costs because that patient has been hospitalized for longer and is in care longer than perhaps should have been necessary.” [Interview 16, healthcare provider]

^1^*QA* = Quadruple Aim; *P* = Improving population health; *Q* = Improving quality of care and patient experience; *S* = Improving staff experience; *C* = Reducing per capita healthcare costs. Based on the reasoning of the interviewees, we assigned the opportunities to the Quadruple Aim.

#### Phase: referral and diagnosis

- **1. A digital intake for an endoscopy could contribute to better-prepared patients**
- According to most of our interviewees, the intake in preparation for the endoscopy — which currently generally takes place in the hospital — could occur digitally. In a digital intake, patients would fill in a digital questionnaire in the patient portal. Also, patients can receive digital information regarding the endoscopy and the required preparation. Patients would not physically visit the outpatient clinic but prepare from home [see quote from I15]. Three interviewees indicated that their hospital already conducts digital intakes and stated that it is generally rated positive by patients and providers.
  “Because they always have to take another morning off to come to the hospital [for the intake preceding the endoscopy], and then they have to take another day off for the endoscopy, and they can, let’s say, do this [a digital intake] in between.” [Interview 15, healthcare provider].

#### Phase: treatment

- **2. Online information services could better inform patients about treatment options**
- Online information services could provide more extensive information about the (dis)advantages and risks of various treatment options in addition to the information provided during the consultations. Digital information could be offered in various forms, such as an online decision aid, educational videos, or online platforms where patients who have undergone the treatment share their experiences. This information can be used as input for the doctor and patient discussion.
- **3. Digital communication and online information services could facilitate treatment preparation**
- Digital video consultations are already being used but could be scaled up to replace regular in-person consultations in preparation for treatment. Patients could be offered information videos via the patient portal. The information could be used to prepare for the consult or to look up information afterward. Also, interviewees indicated that patients should be able to chat with a healthcare provider to ask remaining questions about the treatment.
- **4. A digital application could support patients in the prehabilitation process**
- During the prehabilitation program, interviewees see an opportunity for digital care to support patients. Standard care involves a personalized program — based on an intake interview — designed by care providers such as a doctor, a dietician, and a physiotherapist. A digital application offering information, daily goals, and reminders could complement such standard care. Patients could fill in their results in the application, which monitors whether patients achieve their (daily) goals and provides personalized advice. The results can be shared with the healthcare provider. Some interviewees mentioned that only a monitoring function would suffice for some patients.

#### Phase: aftercare

- **5. Remote wound and stoma care could reduce hospital visits and consultations**
- Interviewees indicated that patients should be able to upload a photo of their wound or (skin) effects of the stoma, from home, in the patient portal [see quote from I5]. The doctor or wound nurse could then remotely assess and give advice. In addition, patients could also ask them questions via the patient portal. In addition, a healthcare provider could make video calls with home care to supervise wound and stoma care directly.
  “Patients sometimes ask, ‘my wound is getting a little red; what is normal?’. It would be easy to send images of what it looks like.” [Interview 5, healthcare provider].
- **6. Patient telemonitoring in the post-operative phase could increase the chance of early complication recognition**
- Patients could wear a smart patch that measures health values such as heart rate and temperature. In addition, patients could fill out daily questionnaires about pain, eating and drinking, mobility, and psychological stress in a digital application on their mobile phones. According to some interviewees, patients could be telemonitored for a certain period after surgery, detecting patient deterioration as early as possible. Daily monitoring of patients’ health values could also occur within the hospital.
- **7. Telemonitoring for prolonged patient monitoring after treatment**
- Interviewees mentioned that healthcare providers could telemonitor patients’ physical and mental conditions. Patients would monthly fill in a digital questionnaire about weight, constipation complaints, blood in the stool, and psychosocial load. The results are shared with healthcare providers. Patients would also get blood test reminders. The telemonitoring data could guide the choice for (the frequency of) (digital) contact, allowing for a risk-based approach to follow-up care. One of the interviewees mentioned the Assessment of Burden of Colorectal Cancer tool, a monitoring tool consisting of digital questionnaires that patients fill in regularly about the experienced burden of CRC and assessment of lifestyle parameters, supporting aftercare [29].

#### Phase: palliative care

- **8. Digital information about physical and psychological complaints could support patients**
- Patients in the palliative phase could receive digital information about complaints in this phase. The information could be provided through videos about common complaints in the patient portal or an online decision aid.
- **9. A digital Advance Care Planning questionnaire could support joint decision-making**
- Patients could fill in a digital Advance Care Planning questionnaire to map out wishes and needs and decide on possible further treatment or stopping treatment. The questionnaire could also be used as input for conversations with healthcare providers.

#### Care pathway phase transcending

- **10. Digital consultation hours or a chat could unburden staff**
- Patients could ask questions during digital walk-in consultation hours with nurses. This way, there is one central point of contact. Alternatively, patients could ask questions via a chat function in the patient portal, to which a doctor or nurse responds asynchronously. Some interviewees mentioned that specific digital applications that enable video communication, chatting, and exchanging documents between patients and healthcare providers already exist (i.e., the Dutch application *BeterDichtbij*) [30].
  “I know that almost all patients have a nurse practitioner as their contact person. There are, within the hospital, simply very few of these for the number of patients they should accompany. So I would envision a telephone consultation hour, or someone on a chat, with nursing experience, who could answer those kinds of questions.”
- **11. Digital questionnaires in preparation for a consultation could facilitate patients to report complaints or ask questions**
- Interviewees indicated that patients could fill in digital questionnaires via the patient portal to prepare for consultations. Consultation questionnaires at the beginning of the care path would contain questions about auto-anamnesis. Later in the care pathway, consultation questionnaires would deal with positive psychology, well-being, value-driven care, and psychosocial health themes.

#### Hospital care setting transcending

- **12. More efficient digital data exchange improves the availability of patient data for healthcare providers and patients**
- In the short term, healthcare providers would wish to have the possibility to, across organizations, retrieve data from external electronic health records (EHRs) of patients with whom they have a treatment relationship. Interviewees indicated they should not have to rely on an external party, such as a hospital or general practitioner, to receive relevant data. In the long term, all healthcare providers and patients should be able to access the same online system to share and retrieve data.

## Discussion

This qualitative study is the first to gather perspectives of healthcare providers and managers on the use of e-health to improve the CRC care pathway and contribute to the Quadruple Aim.

Interviewees indicated that different types of e-health are already being used in the CRC care pathway but should be better exploited. They see many opportunities for e-health to optimize CRC care, for example, in terms of flexibility. However, there are some concerns, such as some patient groups’ inability to use e-health. In every phase of the CRC care pathway, diverse improvement opportunities have been suggested with the help of different e-health technologies. Some opportunities can be applied in multiple phases of the care pathway or outside the medical specialist care setting.

Several findings stand out when reflecting on the results. Part of the opportunities are substitutes for in-person care, such as a digital intake for endoscopy instead of an intake in the hospital. Other opportunities can move or prevent care, such as online information services providing patients with information about treatment options or common side effects. Some opportunities can be easily implemented using existing applications, such as digital communication. Other opportunities, such as increasing efficiency in patient data exchanges, require major system adaptations, for example, legislative changes. Scientific studies already support some of the improvement opportunities mentioned by interviewees. For example, the hypothesis that a digital intake would save healthcare providers time is in line with the results of a study showing that, in preparation for an endoscopy, computer-based education was non-inferior to nurse counseling and led to 79% fewer patient visits to the outpatient clinic [31].

Identifying improvement opportunities is the first essential step to improving the CRC care pathway. The identified opportunities are yet to be prioritized based on scientific evidence and other factors such as available budget. Also, there are several prerequisites vital to realizing the improvement opportunities. Interviewees mentioned, for example, changes in budget allocations, workplace culture, and privacy legislation. Some of the interviewees’ hospitals already use some improvement opportunities mentioned in their local healthcare setting. Stakeholders involved in CRC care should use the lessons learned from these local practices (e.g., experiences of hospital staff with a digital intake for an endoscopy) to increase their understanding of how to scale these initiatives.

The strength of this study lies in the use of qualitative methods to obtain a clear understanding of the different perspectives of healthcare managers and staff on how e-health can contribute to improving the CRC care pathway. The independent coding of the data by two researchers improves the validity of this study. Some limitations should also be mentioned. First, although we applied a purposive sampling method, healthcare providers and managers who favor or have a clear vision of using e-health might have been more likely to participate. This could have biased the results as interviewees probably expressed less critical remarks and objections to using e-health. However, this is less relevant as the study aimed to identify improvement opportunities rather than to gather all different visions on e-health in the CRC pathway. Secondly, as we only held individual interviews and no focus groups, healthcare providers and managers from different local practices could not exchange views and discuss disagreements, which could have further nuanced the results. Several medical specialists were included in the sample, namely, gastroenterologists and colorectal surgeons. However, we could not recruit oncologists, which may have led to a gap in our findings. Furthermore, as our scope focused on the medical specialist care setting, we did not include the perspectives of healthcare providers working outside the hospital, such as general practitioners. Finally, we used a relatively small sample of 17 participants. However, since data saturation was reached at this point, the findings in this report are presumed to be (largely) generalizable to the target population of this study.

Future research should extend the current study’s findings to include the perspectives of other relevant stakeholders, such as patients and other parties involved in CRC care, for example, general practitioners, psychologists, and dieticians. Secondly, it would be interesting to apply this study method to different types of cancer care to determine to what extent current results are generalizable to other types of (cancer) care.

## Conclusion

This study showed that there are many promising opportunities for e-health to contribute to improving cancer care. To take the next step forward, the perspectives of other stakeholders must be examined, the identified opportunities should be adequately prioritized, and the requirements for successful implementation should be correctly mapped out. This study method can be used for research among other stakeholders and in different healthcare settings to identify how e-health would add value to improvement opportunities that contribute to the Quadruple Aim.

## Supplementary Information

Below is the link to the electronic supplementary material.

Supplementary file1. Overview of usage of and experiences with different e-health categories among the interviewees. (DOCX 24 KB)

## Data Availability

The data that support the findings of this study are available from the corresponding author on reasonable request.
