# Supplementary material for: Improving the colorectal cancer care pathway via e-health: a qualitative study among Dutch healthcare providers and managers
Source: Support Care Cancer. 2023 Mar 6;31(4):203. doi: 10.1007/s00520-023-07653-2 (PMC9986036; doi:10.1007/s00520-023-07653-2)
Supplement: Supplementary file 1 — Supplementary file1. Overview of usage of and experiences with different e-health categories among the interviewees. (DOCX 24 KB) [file 520_2023_7653_MOESM1_ESM.docx]

**Table 1**. Overview of usage of and experiences with different e-health categories among the interviewees.

| **Category** | **Usage and attitudes** |
| --- | --- |
| Digital communication | Nearly all interviewees use digital communication. The usage of digital communication tools increased during the COVID-19 crisis. Some mentioned digital communication fits well with specific stages of the CRC care pathway, such as aftercare and less with other stages, such as diagnosis. The advantages mentioned multiple times were that there is no travel time and family can join more easily. A few interviewees mentioned disadvantages, such as providing support and running late during a digital consult being more complicated. |
| Patient portal | All interviewees stated that patient portals are offered to patients primarily for viewing appointments, results and digital information. Multiple interviewees mentioned the disadvantage that patients can already see results before having a doctor explain them. A possible solution is the delayed release of some information. |
| Online information services | Online information services with information about the disease, diagnosis and treatment are widely used, often through internal initiatives, sometimes by reference to external information. Some were already very satisfied with digital information, others saw opportunities to expand it. |
| Electronic Health Record (EHR) and Electronic data exchange | All interviewees use the EHR. Data exchange between EHRs often takes place digitally, allowing for a smooth exchange of electronic images. However, the different systems used by the various healthcare providers are not always compatible, thereby hindering digital exchange. It is also stated that adding scanned documents is sometimes complicated and that user-friendliness could be improved. |
| Digital questionnaires | Some hospitals use digital questionnaires in preparation for a consultation. A few of the hospitals (*n*=3 out of 13) are already using digital questionnaires as an alternative to an in-person consultation that prepares for an endoscopy. Generally, requesting PROMS^1^ and PREMS^2^ occurs digitally. |
| Telemonitoring and self-monitoring | Some healthcare providers saw potential to apply digital monitoring, but its use is still limited. Interviewees noted that telemonitoring could enable patients to go home earlier while still being monitored at home, shortening hospital submissions. |
| Personal Health Record (PHR) | PHRs were not mentioned often. Interviewees that did mention PHRs indicated that the application is still in its infancy. |
| Decision support software | The use of decision support software is minimal. However, multiple interviewees indicated a potential for future use of decision support software. For example, to support identifying patients at risk and providing treatment advice. A few healthcare providers indicated being unfamiliar with the technology and its possibilities. |

1) Patient Reported Outcome Measures; 2) Patient Reported Experience Measures
